# Supplementary material for: High-throughput Kinetics using capillary Electrophoresis and Robotics (HiKER) platform used to study T7, T3, and Sp6 RNA polymerase misincorporation
Source: PLoS One. 2024 Dec 2;19(12):e0312743. doi: 10.1371/journal.pone.0312743 (PMC11611218; doi:10.1371/journal.pone.0312743)
Supplement: S1 Fig — (PDF) [file pone.0312743.s001.pdf]

S1 Fig

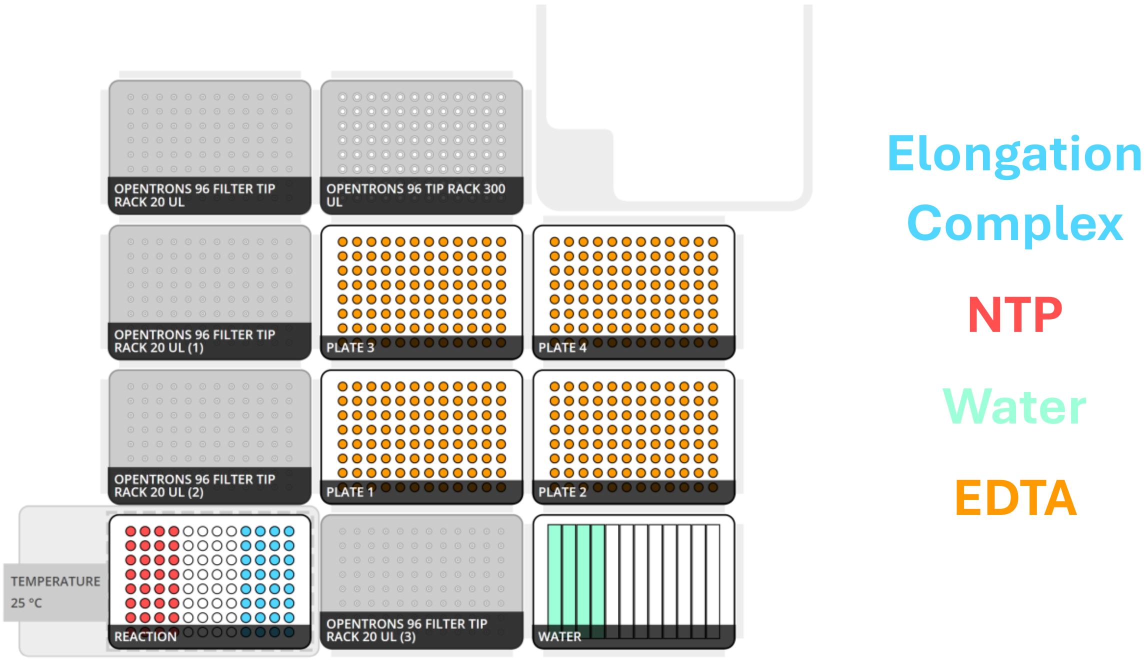

General reagent layout for OT-2 deck when using HiKER to perform nucleotide addition assays. Plate 1 – Plate 4 contains sample collection plates containing excess EDTA to quench the reaction. The reaction plate contains two different groups of reagents. The red left hand side contains the individual NTP solutions in separate wells. The blue right hand side group contains the elongation complex scaffolds.
