# Supplementary material for: High-throughput Kinetics using capillary Electrophoresis and Robotics (HiKER) platform used to study T7, T3, and Sp6 RNA polymerase misincorporation
Source: PLoS One. 2024 Dec 2;19(12):e0312743. doi: 10.1371/journal.pone.0312743 (PMC11611218; doi:10.1371/journal.pone.0312743)
Supplement: S1 Table — (PDF) [file pone.0312743.s002.pdf]

S2 Table

Supplemental Table: Misincorporation Formation Kinetics of T7, T3, and Sp6 RNA Polymerase

T7 RNAP			T3 RNAP			Sp6 RNAP		
	k_{obs} (min^{-1})	Amp		k_{obs} (min^{-1})	Amp		k_{obs} (min^{-1})	Amp
**dA:rA**	0.13 ± 0.03	0.13 ± 0.01	**dA:rA**	2 ± 1	0.083 ± 0.007	**dA:rA**	0.07 ± 0.02	0.17 ± 0.02
**dA:rG**	ND	ND	**dA:rG**	ND	ND	**dA:rG**	0.17 ± 0.05	0.04 ± 0.01
**dA:rC**	0.15 ± 0.01	0.37 ± 0.01	**dA:rC**	0.15 ± 0.02	0.42± 0.02	**dA:rC**	0.07 ± 0.01	0.37 ± 0.03
**dA:rU**	> 5	0.73 ± 0.01	**dA:rU**	> 5	0.97 ± 0.01	**dA:rU**	> 5	0.95 ± 0.01
**dG:rA**	0.15 ± 0.02	0.14 ± 0.01	**dG:rA**	ND	ND	**dG:rA**	0.04 ± 0.02	0.17 ± 0.05
**dG:rG**	ND	ND	**dG:rG**	ND	ND	**dG:rG**	ND	ND
**dG:rC**	> 5	0.83 ± 0.01	**dG:rC**	> 5	0.98 ± 0.01	**dG:rC**	> 5	0.96 ± 0.01
**dG:rU**	0.07 ± 0.01	0.16 ± 0.01	**dG:rU**	0.06 ± 0.03	0.22 ± 0.06	**dG:rU**	0.001 ± 0.02	0.5 ± 0.9
**dC:rA**	0.82 ± 0.09	0.75 ± 0.01	**dC:rA**	1.03 ± 0.05	0.53 ± 0.01	**dC:rA**	0.78 ± 0.02	0.16 ± 0.01
**dC:rG**	> 5	0.88 ± 0.01	**dC:rG**	> 5	0.96 ± 0.01	**dC:rG**	> 5	0.94 ± 0.01
**dC:rC**	1.8 ± 0.2	0.14 ± 0.01	**dC:rC**	1.4 ± 0.3	0.13 ± 0.01	**dC:rC**	0.6 ± 0.1	0.09 ± 0.01
**dC:rU**	1.4 ± 0.2	0.77 ± 0.01	**dC:rU**	1.3 ± 0.1	0.67 ± 0.01	**dC:rU**	0.26 ± 0.02	0.81 ± 0.02
**dT:rA**	> 5	0.86 ± 0.01	**dT:rA**	> 5	0.94 ± 0.01	**dT:rA**	> 5	0.92 ± 0.02
**dT:rG**	0.11 ± 0.01	0.51 ± 0.01	**dT:rG**	0.13 ± 0.01	0.38 ± 0.01	**dT:rG**	0.04 ± 0.01	1.0 ± 0.2
**dT:rC**	ND	ND	**dT:rC**	ND	ND	**dT:rC**	0.10 ± 0.05	0.05 ± 0.01
**dT:rU**	0.23 ± 0.02	0.70 ± 0.01	**dT:rU**	0.18 ± 0.01	0.75 ± 0.01	**dT:rU**	0.20 ± 0.01	0.81 ± 0.01
