# Supplementary material for: High-throughput Kinetics using capillary Electrophoresis and Robotics (HiKER) platform used to study T7, T3, and Sp6 RNA polymerase misincorporation
Source: PLoS One. 2024 Dec 2;19(12):e0312743. doi: 10.1371/journal.pone.0312743 (PMC11611218; doi:10.1371/journal.pone.0312743)
Supplement: S2 Table — (PDF) [file pone.0312743.s003.pdf]

S3 Table

Supplemental Table: T7, T3, and Sp6 RNA Polymerase Extension from a Mismatch Kinetics								
T7 RNAP			T3 RNAP			Sp6 RNAP		
	k_{obs} (min^{-1})	Amp		k_{obs} (min^{-1})	Amp		k_{obs} (min^{-1})	Amp
**dA:rA**	1.3 ± 0.2	0.13 ± 0.01	**dA:rA**	0.29 ± 0.06	0.76 ± 0.04	**dA:rA**	0.07 ± 0.07	0.06 ± 0.03
**dA:rG**	0.2 ± 0.1	0.07 ± 0.01	**dA:rG**	ND	ND	**dA:rG**	ND	ND
**dA:rC**	0.06 ± 0.01	0.84 ± 0.08	**dA:rC**	0.34 ± 0.02	0.95 ± 0.02	**dA:rC**	0.03 ± 0.01	0.7 ± 0.3
**dA:rU**	> 5	0.74 ± 0.01	**dA:rU**	> 5	0.96 ± 0.01	**dA:rU**	0.26 ± 0.04	0.84 ± 0.03
**dG:rA**	ND	ND	**dG:rA**	slope = 0.006 ± 0.001		**dG:rA**	ND	ND
**dG:rG**	0.04 ± 0.03	0.2 ± 0.1	**dG:rG**	ND	ND	**dG:rG**	ND	ND
**dG:rC**	2.4 ± 0.6	0.80 ± 0.02	**dG:rC**	> 5	0.97 ± 0.01	**dG:rC**	0.42 ± 0.04	0.89 ± 0.02
**dG:rU**	0.52 ± 0.04	0.15 ± 0.01	**dG:rU**	0.11 ± 0.01	1.00 ± 0.02	**dG:rU**	0.21 ± 0.06	0.15 ± 0.01
**dC:rA**	0.6 ± 0.4	0.015 ± 0.002	**dC:rA**	0.22 ± 0.04	0.54 ± 0.03	**dC:rA**	slope = 0.004 ± 0.001	
**dC:rG**	> 5	0.80 ± 0.01	**dC:rG**	> 5	0.95 ± 0.01	**dC:rG**	0.32 ± 0.02	0.87 ± 0.01
**dC:rC**	0.03 ± 0.01	1.0 ± 0.3	**dC:rC**	slope = 0.028 ± 0.001		**dC:rC**	slope = 0.011 ± 0.001	
**dC:rU**	0.6 ± 0.2	0.08 ± 0.01	**dC:rU**	0.45 ± 0.07	0.87 ± 0.03	**dC:rU**	0.06 ± 0.02	0.21 ± 0.05
**dT:rA**	3.3 ± 0.5	0.69 ± 0.01	**dT:rA**	> 5	0.93 ± 0.01	**dT:rA**	0.8 ± 0.1	0.76 ± 0.03
**dT:rG**	0.06 ± 0.02	0.4 ± 0.1	**dT:rG**	1.1 ± 0.2	0.61 ± 0.02	**dT:rG**	slope = 0.012 ± 0.001	
**dT:rC**	0.04 ± 0.01	1.1 ± 0.2	**dT:rC**	0.12 ± 0.01	1.06 ± 0.02	**dT:rC**	slope = 0.012 ± 0.001	
**dT:rU**	0.45 ± 0.07	0.19 ± 0.01	**dT:rU**	0.40 ± 0.04	0.89 ± 0.02	**dT:rU**	0.17 ± 0.04	0.08 ± 0.01
