# Supplementary material for: High-throughput Kinetics using capillary Electrophoresis and Robotics (HiKER) platform used to study T7, T3, and Sp6 RNA polymerase misincorporation
Source: PLoS One. 2024 Dec 2;19(12):e0312743. doi: 10.1371/journal.pone.0312743 (PMC11611218; doi:10.1371/journal.pone.0312743)
Supplement: S1 File — (ZIP) [file pone.0312743.s004.zip › S4 HiKER Code.pdf]

**OT-2 HiKER Time Course Script**
**Written in Python**

```
from opentrons import protocol_api
from opentrons.types import Point
import math

metadata = {
   'protocolName': '12.08.2023  P1-P4 Time Courses',
   'author': 'Zach <zcarter@neb.com>',
   'apiLevel': '2.14'
}
#Performs multiple RNAP misincorporation experiments (for single plate, use RNAP Single protocol) in
sequence
#Place NTPs in first x rows, place enzyme in last x rows
#Will stop after 2 plates to prompt trash removal

#Number of Experiments (max 4, min 2)
exps = 4
#Pick 12 time points (in minutes)
timepoints = [0, 0.01, 0.5, 1, 2, 5, 7.5, 10, 12.5, 15, 17.5, 20]
def run(ctx):

   #SET-UP

   #load pipettes + tips
   #300 ul pipette and tips [RIGHT MOUNT, SLOT 11]
   tips300 = [ctx.load_labware("opentrons_96_tiprack_300ul", 11, "Tips300")]
   m300 =ctx.load_instrument('p300_multi_gen2', 'left', tip_racks = tips300)
   #20 ul pipette and tips [LEFT MOUNT, SLOTS 7, 4, 10, 2]
   tips20 = [ctx.load_labware("opentrons_96_tiprack_20ul", 7, "Tips20A"),
ctx.load_labware("opentrons_96_tiprack_20ul", 4, "Tips20B")]
   if exps >= 3:
      tips20 = tips20 + [ctx.load_labware("opentrons_96_tiprack_20ul", 10, "Tips20C")]
      if exps == 4:
         tips20 = tips20 + [ctx.load_labware("opentrons_96_tiprack_20ul", 2, "Tips20D")]
   m20 = ctx.load_instrument('p20_multi_gen2', 'right', tip_racks = tips20)

   #temp module [SLOT 1, 25C]
   temp_mod = ctx.load_module("temperature module gen2", 1) #[DO NOT CHANGE SLOT, ROBOT WILL
COLLIDE DURING CALIBRATION]
   react = temp_mod.load_labware('vwr_96_aluminumblock_100ul', label = "Reaction")  #Change to
'vwr_96_aluminumblock_100ul' to use vwr 100 uL pcr plate on temp mod
   temp_mod.set_temperature(celsius=25)

   #load plates [SLOTS 5, 6, 8, 9]
   finalA = ctx.load_labware('nestedcemult1_96_wellplate_300ul', 5, "FinalPlateA") #Change to
'nestedcemult1_96_wellplate_300ul' to use nested CE plate on biorad 96 well plate
   finalB = ctx.load_labware('nestedcemult1_96_wellplate_300ul', 6, "FinalPlateB") # ''
   FinalPlates = [finalA, finalB]
   if exps >= 3:
      finalC = [ctx.load_labware('nestedcemult1_96_wellplate_300ul', 8, "FinalPlateC")] # ''
      FinalPlates = FinalPlates + finalC
```

```
    if exps >= 4:
        finalD = [ctx.load_labware('nestedcemult1_96_wellplate_300ul', 9, "FinalPlateD")] # ''
        FinalPlates = FinalPlates + finalD

#water set up [SLOT 3]
reservoir = ctx.load_labware('nest_12_reservoir_15ml', 3,"Water")
water = [reservoir.wells()[n] for n in range(exps)] #Fill corresponding number of wells from L to R with water

#liquid set up
NTP = [react.rows()[0][n] for n in range(exps)] #Put NTP in first x rows
Enzyme = [react.rows()[0][n+8] for n in range(exps)] #Put enzymes in x rows, starting from row 9
FinalWells = []
for n in range(exps):
    FinalWells = FinalWells + [FinalPlates[n].rows()[0]] #Create list of wells in CE plates

#DEFINE FUNCTIONS
#time point function
#Old method that doesn't work with trash shoot
#def taketp(rxn,tp):
 #   m20.transfer(5, Enzyme[rxn], FinalWells[rxn][tp], mix_after = (2,10))


center_trash_location = ctx.fixed_trash['A1']
adjusted_trash = center_trash_location.top(z=55).move(Point(x=65, y=5))


#time point function
#Method of collecting time points that allows using the ramp

def taketp(rxn,tp,trash_location=adjusted_trash):
    m20.pick_up_tip()
    m20.aspirate(5,Enzyme[rxn])
    m20.dispense(5,FinalWells[rxn][tp])

    #One Mixing Step
    #m20.aspirate(10, FinalWells[rxn][tp], rate = 1)
    #m20.dispense(10, FinalWells[rxn][tp], rate = 1)
    #m20.drop_tip(trash_location)

    #unmute for Multiple Mixing Steps
    for rep in range(2):
        m20.aspirate(10, FinalWells[rxn][tp], rate = 1)
        m20.dispense(10, FinalWells[rxn][tp], rate = 1)
    m20.drop_tip(trash_location)




#master function to run assay
def runplate(tps, plate,trash_location=adjusted_trash):
    #time point 0
```

```
    #m20.transfer(2, Enzyme[plate], FinalWells[plate][0], mix_after = (2,7))
    m20.pick_up_tip()
    m20.aspirate(2,Enzyme[plate])
    m20.dispense(2,FinalWells[plate][0])
    for rep in range(1):
      m20.aspirate(7, FinalWells[plate][0], rate = 1)
      m20.dispense(7, FinalWells[plate][0], rate = 1)
      #m20.drop_tip(tip_racks["A12"].top(z=10))
    m20.drop_tip(trash_location)

    #begin rxn
    m300.transfer(50, NTP[plate], Enzyme[plate], mix_after = (2, 75))


    #further time points
    for n in range(len(tps)-1):
      ctx.delay(minutes = (tps[n+1]-tps[n]))
      taketp(plate,n+1)

  #BEGIN PROTOCOL
  for n in range(exps):
    runplate(timepoints,n,adjusted_trash)
    #if n == 1:
     #   ctx.pause('Empty Trash')

  #dilute with water
  for m in range(exps):
    m300.distribute(100, water[m], [FinalWells[m][n].top() for n in range(len(FinalWells[m]))], new_tip = 'once',
disposal_volume = 5)

  #deactivate heat module
  temp_mod.deactivate()
```

**HiKER Exact Time Point Run Log Processing Script**
**Written in Python**

```
import json
from datetime import datetime
import itertools
#Put in name of run log file
with open("11.22.2023 Exp1 RunLog.json", "r") as log:
  run = json.load(log)
Commands = run["commands"]["data"]
point_sample = 0
point_plate = 0

#Enter volume removed for each  time points (must be different than amount used to initiate reaction)
tpvol = 10

#Enter number of 96 well plates
Plates = 4
```

```
#Function which converts time readout to min
def get_min(time_str):
   # split in hh, mm, ss
   hh, mm, ss = time_str.split(':')
   return int(hh)*60 + int(mm)  + float(ss)/60


#Find Time Point 0 in run log file
PlateStarts = {}
for n in range(len(Commands)):
   if Commands[n]["commandType"] == 'dispense':
    if Commands[n]["params"]['volume'] == 50:
      PlateStarts[point_plate] = Commands[n]["completedAt"]
      point_plate = point_plate + 1


#Find Time Points 1+ in run log file
Times = {}
for n in range(len(Commands)):
   if Commands[n]["commandType"] == 'dispense':
      if Commands[n]["params"]['volume'] == tpvol:
         Times[point_sample] = Commands[n]["completedAt"]
         point_sample = point_sample + 1


#Convert Times to datetime
for n in Times:
   Times[n] = Times[n][:len(Times[n])-6]
   Times[n] = datetime.strptime(Times[n], '%Y-%m-%dT%H:%M:%S.%f')

#print(Times)
#print('\n\n')

#Index times for each plate to start
for n in PlateStarts:
   PlateStarts[n] = PlateStarts[n][:len(PlateStarts[n])-6]
   PlateStarts[n] = datetime.strptime(PlateStarts[n], '%Y-%m-%dT%H:%M:%S.%f')

#print(PlateStarts)


#Find True time points from when plate started
TimesBetween = []
for i in range(Plates):
   for n in range(11*i,11*i+11):
      TimesBetween = TimesBetween + [Times[n]-PlateStarts[i]]
```

```
#Print Difference between timepoints
TimeStrs = {}
for n in range(len(TimesBetween)):
   TimeStrs[n] = [str(TimesBetween[n])]

#print(PlateStarts)
#print(TimeStrs)

#Make CSV output file. Result is one column with 11 reaction time points for each plate. The first is exculded as it's zero.
#The reaction time stars back to zero when a new plate is started.
with open('Run Times.csv', 'w') as f:
   for key in TimeStrs.keys():
      f.write("%s, %s\n" % (key, get_min(''.join(TimeStrs[key]))))
```

**HiKER Exact Time Point Run Log Processing Script**
**Written as Jupyter Notebook**

```
#Importing Needed Tools

import numpy as np
from scipy.optimize import curve_fit
import matplotlib.pyplot as plt

#Import Data from Excel and Create Data Frame

import pandas as pd
data_df = pd.read_excel(r"C:\Users\zcarter\HiKER High-Throughput Calculation of kobs Script\11.22.2023 P4 Data_Analysis.xlsx",sheet_name ="Fin_Mat")


#Convert Data Frame to Arrary

exp_mat = pd.DataFrame(data_df).to_numpy()





#Label Individual Time Courses

t1 = exp_mat[:,0]
```

```
y1 = exp_mat[:,1]
t2 = exp_mat[:,2]
y2 = exp_mat[:,3]
t3 = exp_mat[:,4]
y3 = exp_mat[:,5]
t4 = exp_mat[:,6]
y4 = exp_mat[:,7]
t5 = exp_mat[:,8]
y5 = exp_mat[:,9]
t6 = exp_mat[:,10]
y6 = exp_mat[:,11]
t7 = exp_mat[:,12]
y7 = exp_mat[:,13]
t8 = exp_mat[:,14]
y8 = exp_mat[:,15]



# Define function you want to fit your data with

def func(x, k, A):
  return A *(1 - np.exp(-k * x))

#Fit your data

params1, pcov1 = curve_fit(func, t1, y1)
params2, pcov2 = curve_fit(func, t2, y2)
params3, pcov3 = curve_fit(func, t3, y3)
params4, pcov4 = curve_fit(func, t4, y4)
params5, pcov5 = curve_fit(func, t5, y5)
params6, pcov6 = curve_fit(func, t6, y6)
params7, pcov7 = curve_fit(func, t7, y7)
params8, pcov8 = curve_fit(func, t8, y8)



# Report Best Fit Parameters (kobs, Amp) and Generate Best Fit Lines With Experimental Data

print(params1)
print(params2)
print(params3)
print(params4)
print(params5)
print(params6)
print(params7)
print(params8)




plt.scatter(t1, y1)
plt.scatter(t2, y2)
plt.scatter(t3, y3)
```

```
plt.scatter(t4, y4)
plt.scatter(t5, y5)
plt.scatter(t6, y6)
plt.scatter(t7, y7)
plt.scatter(t8, y8)



xsim = np.linspace(0, 28, 150)

plt.plot(xsim, func(xsim, *params1))
plt.plot(xsim, func(xsim, *params2))
plt.plot(xsim, func(xsim, *params3))
plt.plot(xsim, func(xsim, *params4))
plt.plot(xsim, func(xsim, *params5))
plt.plot(xsim, func(xsim, *params6))
plt.plot(xsim, func(xsim, *params7))
plt.plot(xsim, func(xsim, *params8))


#Organize simulations into master array
sim_master_array=np.array([xsim, func(xsim, *params1),xsim, func(xsim, *params2),xsim, func(xsim,
*params3),xsim, func(xsim, *params4),xsim, func(xsim, *params5),xsim, func(xsim, *params6),xsim,
func(xsim, *params7),xsim, func(xsim, *params8)])
sim_master_array=np.transpose(sim_master_array)

#Convert master simulations array into a data frame
Sims_Matrix_DF = pd.DataFrame(sim_master_array,columns=['t1', 'y1', 't2', 'y2', 't3', 'y3', 't4', 'y4', 't5', 'y5', 't6',
'y6', 't7', 'y7', 't8', 'y8'])


#Export Fit Parameters, Experimental Data, and Simulated Data to Excel Sheet

#Orgnize fit parameters into one master array
Params_Matrix_Arr = np.array ([params1,params2,params3, params4, params5, params6, params7,
params8])

#Convert master fit parameters array into a data frame
Params_Matrix_DF = pd.DataFrame(Params_Matrix_Arr, columns=['kobs','Amp'])



with pd.ExcelWriter('Data_Analysis.xlsx') as writer:
   Params_Matrix_DF.to_excel(writer, sheet_name='Fit_Parameters')
   data_df.to_excel(writer, sheet_name='Experimental_Data')
   Sims_Matrix_DF.to_excel(writer, sheet_name='Simulation_Data')
```
