# Supplementary figures and images for: High-throughput Kinetics using capillary Electrophoresis and Robotics (HiKER) platform used to study T7, T3, and Sp6 RNA polymerase misincorporation
Source: PLoS One. 2024 Dec 2;19(12):e0312743. doi: 10.1371/journal.pone.0312743 (PMC11611218; doi:10.1371/journal.pone.0312743)

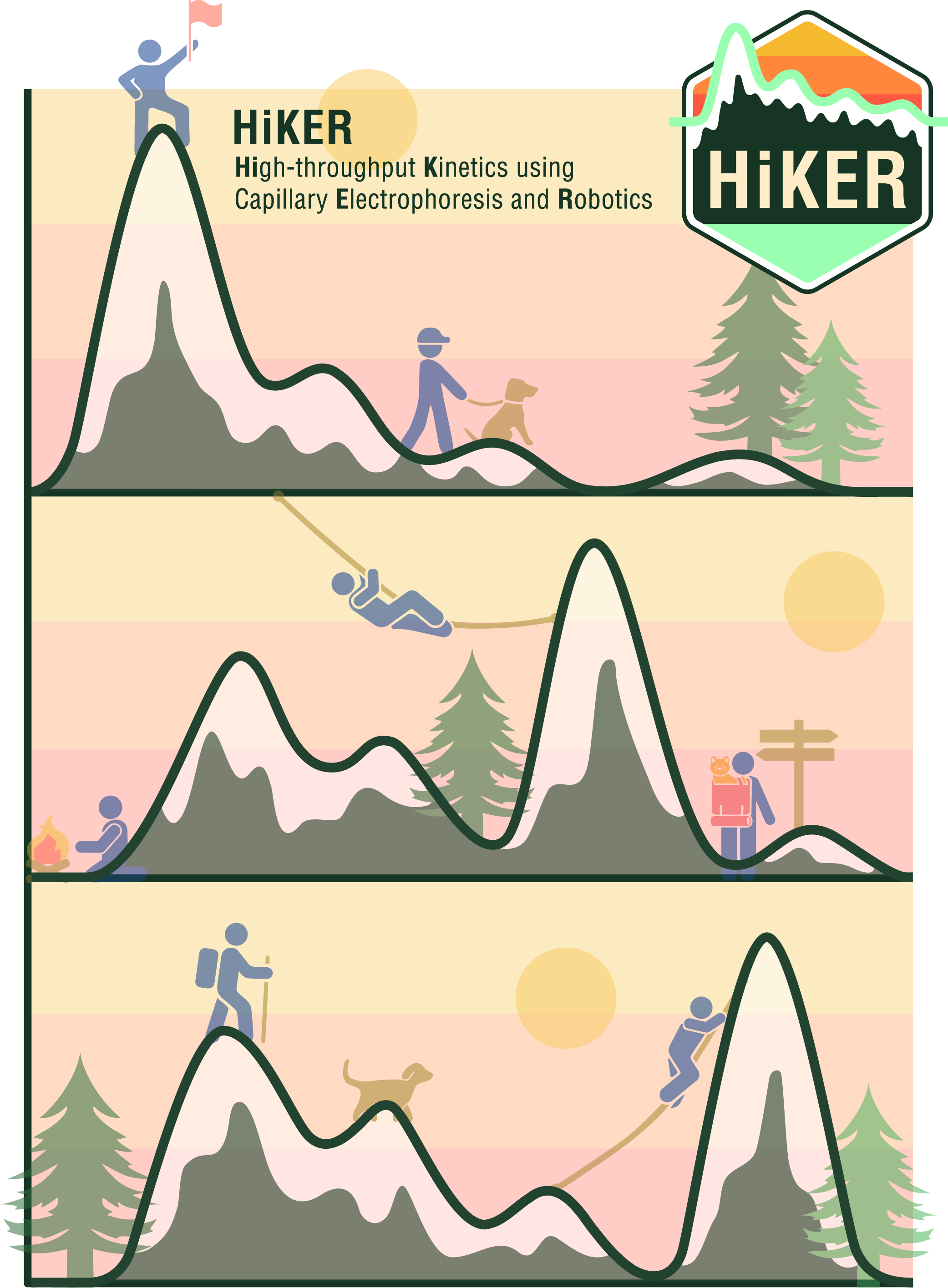

Supplement: S1 Graphical abstract — (TIF) [file pone.0312743.s005.tif]
